# Potential of Using Wood Biomass Ash in Low-Strength Composites

**DOI:** 10.3390/ma14051250

**Published:** 2021-03-06

**Authors:** Ana Baričević, Ivana Carević, Jelena Šantek Bajto, Nina Štirmer, Marija Bezinović, Keti Kristović

**Affiliations:** 1 Department of Materials, Faculty of Civil Engineering, University of Zagreb, Fra Andrije Kačića Miošića 26, 10000 Zagreb, Croatia; ivana.carevic@grad.unizg.hr (I.C.); jelena.santek.bajto@grad.unizg.hr (J.Š.B.); nina.stirmer@grad.unizg.hr (N.Š.); marija.bezinovic95@gmail.com (M.B.); 2 Trames Ltd., Šipčine 2, 20000 Dubrovnik, Croatia; keti.kristovic@trames.hr

**Keywords:** wood biomass ash, hydraulic lime, coal fly ash, historical buildings

## Abstract

Reducing greenhouse gas emissions and dependence on fossil fuels is the cornerstone of all European climate and energy strategies. Consequently, renewable energy sources are becoming more competitive with fossil fuels. The largest source of bioenergy in the European Union is biomass-fired power plants. Therefore, the European coal phase-out strategy led to an increased use of wood biomass as a sustainable fuel, generating large amounts of wood biomass ash (WBA). In the research studies reported so far, WBA has been mainly used in cementitious composites. However, given the similarities between the chemical composition of WBA and hydraulic lime (HL), this research focused on its potential classification as a building lime. Overall, three different sources of fly WBA were considered for the preparation of binders as mixtures of WBA and coal fly ash (CFA) in different ratios. The contribution of each binder mixture on the paste and mortar properties was analyzed based on the chemical composition, setting time, volume stability, and contribution to the mortar strength (compressive and flexural). In general, it can be concluded that the studied binders can meet the criteria of EN 459-1. However, special attention should be paid to the volume deformations and the setting time.

## 1. Introduction

The development of building materials, both in terms of mechanical properties and durability, is challenging in the restoration of historic buildings, as traditional and modern materials are not compatible. Historic buildings have in common that they are altogether made of natural raw materials such as stone and bricks bound with lime mortar. Proper selection of repair materials is essential to achieve long-lasting restoration. Therefore, lime-based materials are necessary to achieve suitable material compatibility.

At the same time, renewables are going mainstream, have irrevocably shed the label “alternative” and are meeting the world’s growing energy needs. This underpins Europe’s plan to achieve climate neutrality by 2050, the backbone of European Green Deal, underpinned by a legally binding target of net zero greenhouse gas emissions [1]. Although climate neutrality is controversial, wood biomass is considered a CO_2_-neutral energy source because it releases almost as much CO_2_ when burned as it absorbs during growth, making it one of the most important biomass sources for energy production in the EU, with a majority share of 59% of all renewables in terms of gross final consumption [2,3,4]. Based on the Global Bioenergy Statistics [5] report for an 18-year period (from 2000 to 2018), it can be seen that renewable energy sources have experienced significant growth and bioenergy (solid biomass, municipal waste, industrial waste, biogases, liquid biofuels) has the largest share in the total primary energy supply of renewable energy in 2018. The largest share of bioenergy is covered by solid biomass sources including wood chips, wood pellets, and traditional biomass sources. The use of bioenergy could make a significant contribution to the design of a low-carbon energy system in the EU, driven by sustainable production and use of wood biomass [2,6]. A bioeconomy based on locally produced biomass can effectively reduce supply chain uncertainty and provide stability—an important cornerstone for Europe’s green recovery after COVID-19 [7,8]. Accordingly, the industrial use of biomass for thermal and electrical energy production is expected to triple by 2035, compared to 2008 levels [9,10]. The current proliferation of wood biomass power plants already results in an immense amount of wood biomass ash (WBA). The combustion of 1 ton of wood biomass produces approximately 3% ash, resulting in a significant amount of this biogenic waste material [11]. The predominant practice in dealing with ash is direct disposal in landfills and use as a soil amendment, often without any monitoring, indicating the urgent need for proper management of WBA [2,12,13,14,15,16,17]. The valorization of WBA as a sustainable substitute for primary raw materials would help to reduce energy and material intensity and intensify the use of alternative materials. With the adoption of the new "Circular Economy Package", which includes the amending Directive 2018/851 on waste and the Directive 2018/850 on waste landfilling, the European Union additionally promotes decreasing waste disposal in landfills [18]. On the other hand, according to Croatian National Environmental register of pollutants, WBA is classified as a waste whose free recycling is currently not possible domestically. In the research studies reported so far, WBA has been identified mainly as a resource substitute in cementitious composites. However, considering the similarities between the chemical composition of WBAs and hydraulic lime (HL), this research focused on its possible classification as building lime and with an effort to strongly emphasize the problem of industrial waste disposal.

Most WBAs are positioned in the CaO-Al_2_O_3_-SiO_2_ ternary diagram in the range between limestone and portland cement [19]. When considering mean values, the following oxides are mainly represented in WBA [11]: CaO (43.03%) > SiO_2_ (22.22%) > K_2_O (10.75%) > MgO (6.07%) > Al_2_O_3_ (5.09%) > P_2_O_5_ (3.48%) > Na_2_O (2.85%) > SO_3_ (2.78%). Basically, WBAs are rich in calcium oxides, and the CaO content can even exceed 70% depending on the type of biomass used [20]. Compared to coal fly ash (CFA), WBA contains more soluble alkalis, sodium and potassium and less aluminum oxides [21]. The chemical composition of WBA is also highly variable and depends on the combustion technology. Therefore, quality control is essential in the production and application of biomass ash, even compared to coal fly ash, which is widely used regardless of the variations in its chemical composition [17,22]. Table 1 shows the requirements for HL according to the standard EN 459-1 [23], i.e., the properties of binders, pastes and mortars that must be met for the material to be classified as HL.

At the same time, the electricity industry produces millions of tons of coal ash every year. In most countries, storage rather than reuse, is the default solution for coal ash management. The American Coal Ash Association (ACAA) reports each year on the production and use of coal ash in the United States, and their data show that about 60% of the coal fly ash produced is reused [24]. Considering that any reuse of CFA is beneficial, this research aims to combine the benefits of using WBA and CFA in the construction sector.

Inspired by a small number of research studies conducted on cementless mortars using WBA [13,22], the overall objective of this research was to produce mortar using WBA and coal fly ash (CFA) and presents a continuation of research reported at [25]. Specific objectives were: (a) selection of suitable WBAs by analyzing their chemical composition, (b) preparation of an alternative binder system by combining WBA and CFA, (c) analysis of the setting rate and volume stability of such systems. The following hypotheses are put forward: (1) it is possible to produce an alternative binder system by choosing the right proportion of WBA and CFA, (2) combustion technology and biomass origin influence the chemical composition of WBA, which is reflected by the variable content of CaO, SiO_2_, and Al_2_O_3_ and the amount of free calcium hydroxide, thus directly affecting the applicability of WBA as hydraulic lime.

## 2. Materials and Methods

The experimental part of the research aimed to determine a suitable type of WBA for the production of hydraulic lime, taking into account the chemical composition of WBA. For this purpose, it was necessary to determine the appropriate proportion of WBA and CFA that would ensure the hardening of these binder systems.

### 2.1. Wood Biomass and Coal Fly Ash

The wood biomass fly ash (WBA) used came from three different wood biomass plants in Croatia and was combined with coal fly ash (CFA) obtained from a coal-fired power plant in Croatia. The general characteristics of the plants, i.e., combustion type and temperature, and the type of wood biomass are given in Table 2. The combustion technologies and the type of biomass used affect the chemical composition of the WBA [13,22], which directly affects the possibility of mortar hardening and thus the applicability of WBA as a lime substitute.

Samples WBA1 and WBA3 were collected in January, while sample WBA2 was collected in April of the same year. Coal fly ash (CFA), which complies with EN 450-1 [26], was obtained from a coal-fired power plant in Croatia.

A total of 8 binder blends were prepared, as a mixture of WBA and CFA in the following ratios: 100:0, 75:25, 50:50, and 25:75.

### 2.2. Paste and Mortar Production

For the preparation of the paste mixtures, 500 g of binder blends and potable water were used. The amount of water was determined to achieve a standard consistency according to EN 196-3 [27]. Mortars were prepared using binder blends, potable water and standard sand according to EN 196-1 [28]. The mass ratio of binder: aggregate was 1:1.5 and the water content was determined to achieve a slump of (185 ± 3) mm as prescribed in EN 459-2. The chemical additive, a polycarboxylate plasticizer, was added to each mix at 1% by mass of the binder. Suggested designations for pastes and mortars were: (a) Pi-100, Pi-75, Pi-50, Pi-25 for pastes, (b) Mi-100, Mi-75, Mi-50 and Mi-25 for mortars, where *i* is the WBA sample number.

Fresh state properties for both, pastes and mortars, were determined immediately after mixing. After casting, the specimens were kept covered at laboratory conditions for 24 h until demolding, to prevent water evaporation. After demolding, the specimens were in mist room at 20 ± 2 °C and RH ≥ 95%, until testing compressive and flexural strength. For capillary absorption test, specimens were kept at 20 ± 2 °C and RH ≈ 65%, according to the EN 1015-18 [29]. Compressive and flexural strength were tested at the age of 7 and 28 days, while capillary absorption was measured after 28 days. Each property was tested on 3 specimens.

### 2.3. Methods

Hydraulic lime (HL) is classified according to the standard EN 459-1 [23]. Conformity with this classification is assessed using the standard methods given in Table 3, in accordance with the standard EN 459-2 [30]. The physical and chemical properties of each type of WBA and CFA were determined prior to mixing the binders to verify that they conform to EN 459-1 [23].

Thermogravimetric analyses (TGA) of CFA and non-sieved WBA were performed using a TGA 55 instrument, with a platinum container filled with approximately 50 mg of powder sample. The temperature range was between 35 and 950 °C at a constant heating rate of 20 °C/min using inert gas (nitrogen, flow rate: 60 mL/min).

The compliance of the tested binders with the criteria listed in Table 1, taking into account the WBA content in the binder, was carried out as follows [22]:

X_i_ = X_CFA_ × (%/100)_CFA_ + X_WBA_ × (%/100)_WBA_ (1)

where:

X_i_ expressed value of observed physical or chemical property
X_CFA_ value of the physical or chemical property for CFA
X_WBA_ value of the physical or chemical property for used WBA
(%/100)_CFA_ content of CFA in the mixture, expressed in percentages
(%/100)_WBA_ content of WBA in the mixture, expressed in percentages

Chemical composition was performed using the Energy Dispersive X-Ray Fluorescence Spectrometer (EDXRF) Nex CG (Rigaku, TX, USA) and determined for all samples in April, while TGA was performed in December, although the authors suggest that characterization should be performed for the same age of specimens. During this period, the WBA samples were stored sealed in the plastic bags.

The conformity of the wood biomass-coal fly ash system as a binder for repair mortar was evaluated according to the methods given in Table 4, in accordance with the standard EN 459-2 [30].

## 3. Results and Discussion

### 3.1. WBA Characterization

**Figure 1:**
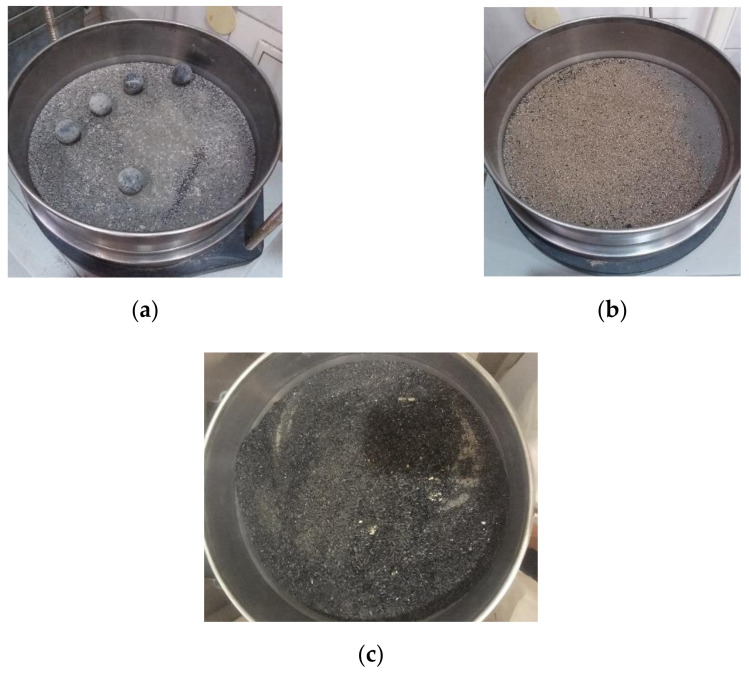
Selective removal of obvious impurities, such as unburnt pieces of wood or coal residues-residues of the sieve 0.25 mm for: (**a**) WBA1 and rubber balls used for sieving; (**b**) WBA2; (**c**) WBA3.

The authors [31] showed that untreated biomass ash has high values of water absorption, high organic matter content, and low density. Therefore, selective removal was used to reduce the negative influence on the binder properties [32]. To determine the particle size distribution, the WBAs were sieved through a 0.25 mm sieve mesh to remove the obvious impurities, such as pieces of unburned wood or coal residues, Figure 1. Rubber balls were used during sieving to ensure proper disintegration of WBA particles. Designation of sieved were WBAs, while non-sieved were WBA.

**Figure 2:**
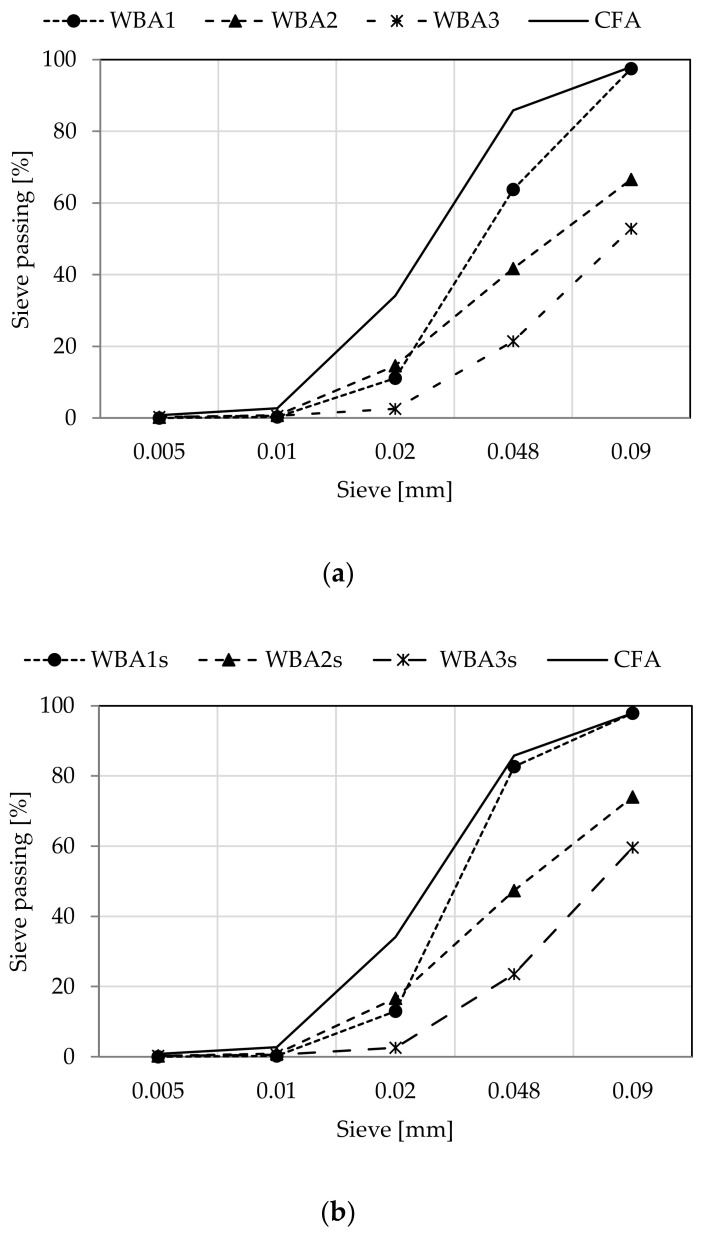
Particle size distribution of (**a**) non-sieved WBAs and CFA; (**b**) sieved WBAs and CFA.

Particle size was determined by air jet method according to EN 459-2 [30], Figure 2. Significant differences in granulometries were found depending on the WBA source. Regardless of post-processing, all WBA samples examined can be specified from smaller to larger particle size as follows: WBA1< WBA2 < WBA3.

According to the physical requirements in EN 459-1 [23], the residue on the sieve 0.09 mm must be less than or equal to 15% of the binder mass. Of the specimens tested, only WBA1 meets the specified criteria with a percentage of particles larger than 0.09 mm of 2.51% for the non-sieved sample, and 2.13% for the sieved sample. The two samples, WBA2 and WBA3, do not meet the established criteria as the percentage of particles larger than 0.09 mm is 33.41 and 47.18% for the non-sieved samples, and 25.95 and 40.40% for the sieved samples. Screening through a 0.25 mm sieve reduces the percentage of larger particles and impurities by 17 ± 4% on average.

The chemical analysis of WBAs (before and after sieving) is shown in Table 5. Basically, WBAs are rich in calcium oxides. Based on the results of chemical analysis, calcium oxide (CaO) had a significant contribution to the total oxide content of samples WBA1 and WBA2. The content of oxides CaO, SiO_2_, Al_2_O_3_, and Fe_2_O_3_ is within the usual values for hydraulic lime (HL) [33,34]. On the other hand, the CaO content in sample WBA3 was lower by a factor of 2 to 3 compared to samples WBA2 and WBA1. Several parameters, such as the type of biomass, the combustion technology, the temperature of biomass combustion, the place of collection and storage [17,20,35,36] influence the characterization of WBA. Based on the general characteristics of the plants listed in Table 2, no conclusion can be drawn about the input parameter of the power plant causing higher SiO_2_ content for the WBA3 sample. The increased content of pozzolanic oxides (60.34%) in WBA3 suggests that it is chemically related to CFA rather than HL [26]. It should be noted that sample WBA1 has an excessive amount of sulphate, which can be potentially harmful to lime-based composites [37,38]. The sulphate content in the WBA samples could result from the fertilizer and impurities (additives or contaminants) generated during the processing of the natural biomass [20]. When biomass is burned in the pulverized fuel power plants, sufficiently high sulfur levels may support the formation of alkali sulphates at high temperatures [39].

**Figure 3:**
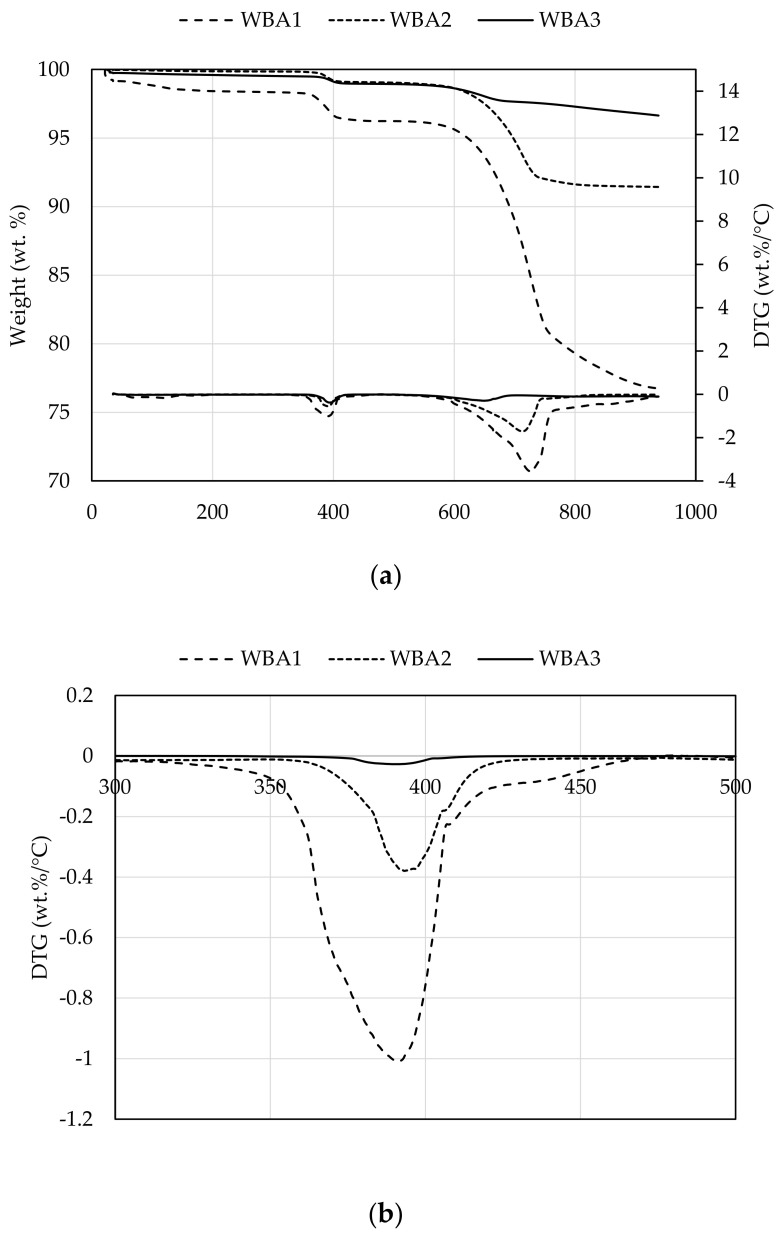
(**a**) TGA and DGT of WBA samples; (**b**) Detailed DTG curve for WBA samples.

Thermogravimetric (TG) analysis and differential thermogravimetry (DTG) of the WBA samples are shown in Figure 3a,b. Overall, three main peaks can be distinguished from the TGA diagram. The mass loss in the range below 200 °C is attributed to moisture evaporation [41], the second peak at 400 °C to portlandite, and the third after 600 °C to the decomposition of calcium carbonate to CaO and CO_2_ [42,43,44]. In all observed samples, there is a large amount of CaCO_3_, especially in WBA1 and WBA2, which is visible by a significant mass decrease at temperatures above 600 °C on TG diagrams. The same was observed by [45], where the mass loss above 650 °C is attributed to the release of carbon dioxide from the thermal decomposition of various carbonates. The amount of carbonate is correlated with the particle size distribution: the finer the particles, the greater the amount of carbonates [46]. Calcium carbonate and Portlandite fraction were calculated from the TGA measurements according to the procedure given in [47]. It can be seen that the WBA1 sample has the highest amount of free CaO, contrary to the results in Table 5. This is explained by the different ages of the WBA samples during the TGA measurements. The aging of the samples possibly contributed to the stabilization of the WBA by carbonation [22,48].

The loss of ignition (LOI) values are within the limits of EN 459-1 [23] for all three WBA types, with a highest LOI (17.7%) determined for the WBA1 sample. The three times higher alkali value (15.3%) for WBA1 sample compared to WBA2 (5.9%) and WBA3 (5.59%) could be attributed to the combustion technology of wood biomass. In pulverized fuel incinerators, peak flame temperatures can be very high, usually around 1600 °C [49], resulting in higher alkali content in the ash [50].

By comparing the results of chemical analysis before and after sieving through a 0.25 mm sieve mesh, minimal differences in the content of each chemical compound are found. According to [51], screening can be used as a simple method to recover a significant portion of the unburned carbon contained in large particles of fly ash from biomass-fired power plants such as the WBA3 sample. Unburnt carbons tend to concentrate in the granulometric fractions with higher particle size, especially in the fly ash from grate furnace combustion [52].

### 3.2. Binder Characterization

Based on Table 5, WBA3 was discarded due to its high content of silica and low content of available lime. These parameters indicate low compatibility of WBA3 and CFA, resulting in delayed hardening and insufficient strengths [53]. Therefore, a total of eight binders were prepared, four for each WBA (WBA1 and WBA2), as a mixture of WBA and CFA in the following ratios: 100:0, 75:25, 50:50, and 25:75. In addition, the contribution of each binder type to the properties of the pastes and the mortars was analyzed using the test methods presented in Table 3.

For a binder to be considered a hydraulic lime substitute, the criteria defined in EN 459-1 [23] must be met, Table 1. Based on the results obtained, all the binders tested met the criteria in terms of SO_3_ content and can be classified as HL 5 based on the available lime content.

**Figure 4:**
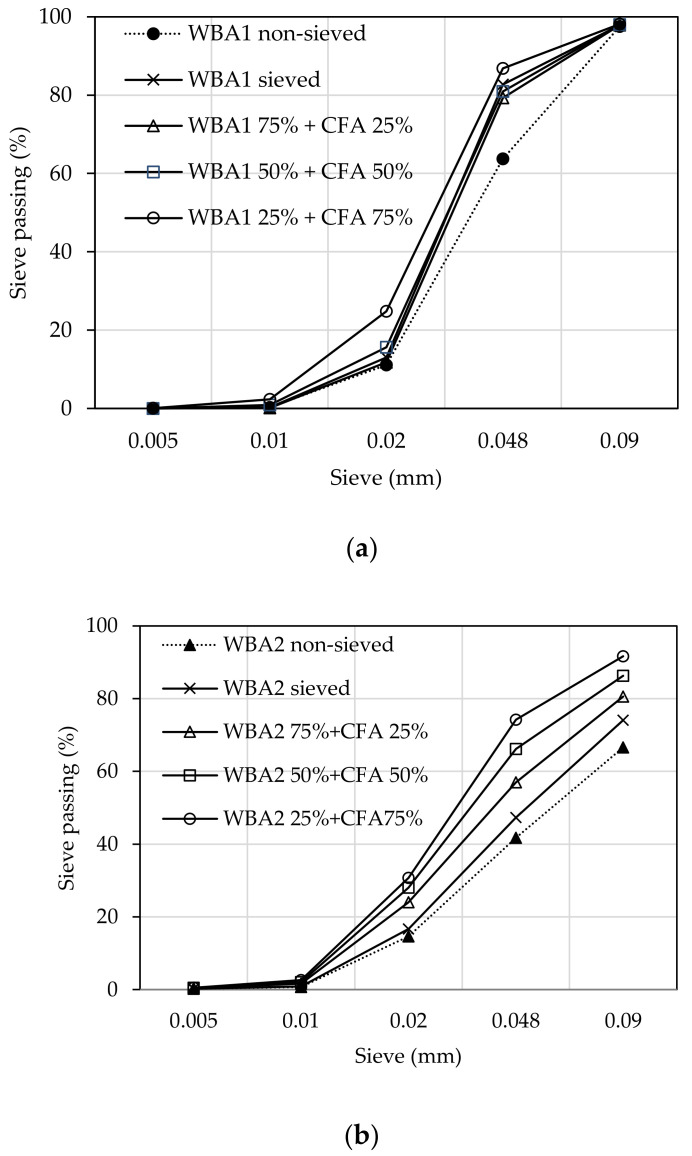
Particle size distribution of; (**a**) binders with WBA1; (**b**) binders with WBA2.

The combination of WBA with CFA resulted in a finer particle size distribution, Figure 4. This is particularly pronounced for binders containing WBA2. The physical requirements for hydraulic lime, given in EN 459-1 [23], require that the residue on the sieve size 0.09 mm is less than or equal to 15% of the binder mass. All binders with WBA1 met the criteria of EN 459-1, while for WBA2 only the binders with the lower content of WBA (WBA 50% + 50% CFA and WBA 25% + 75% CFA) met the criteria.

**Figure 5:**
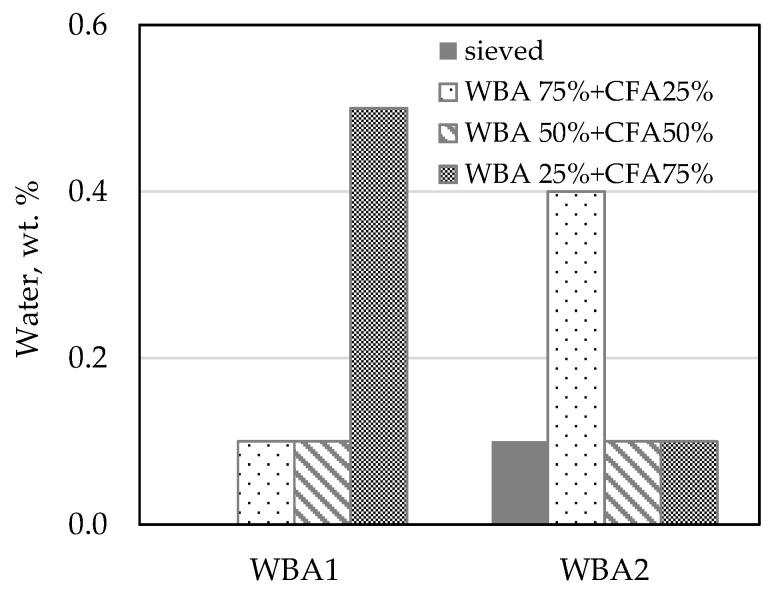
Free water content of WBA binders.

In a hydrated lime (HL), the free water is the moisture bound to the lime particles. Normally, a smaller particle size is expected to have a higher water content [41]. However, in this study, there was no correlation between the amount of free water and the WBA content in the binder blend (Figure 5). The free water content for all binders ranged from 0 to 0.5% by weight of the binder, which satisfy the criteria of the HRN EN 459-1 standard (2%) [23].

### 3.3. Influence of WBA and CFA Binder on Pastes Properties

**Figure 6:**
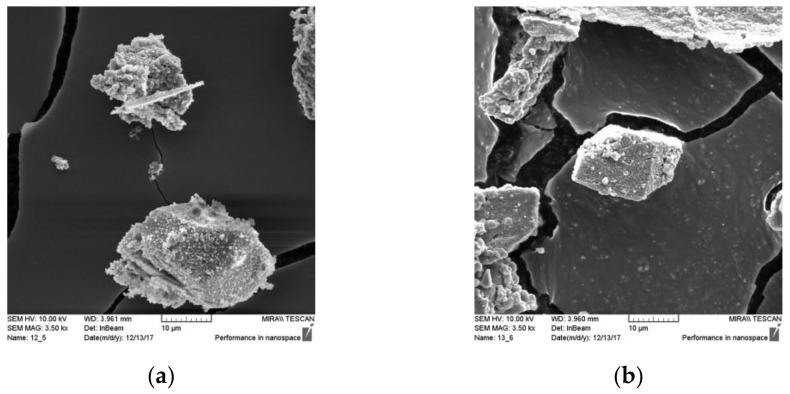
Micrographs of (**a**) WBA1 sample; (**b**) WBA2 sample (scanning electron microscopy magnification, SEM_MAG = 3500×).

Table 6 shows the water requirements for various binder blends, represented by the percentage of coal fly ash needed to achieve standard consistency. Wood biomass ash appears to have a higher water requirement to achieve a similar consistency, so the more WBA added to a mix, the higher the water to binder ratio. This is consistent with previous studies using WBA as a cement replacement [17,22,31,54,55,56,57,58]. The characterization of WBA shows irregular shaped (Figure 6) and highly porous particles with higher specific surface area, along with high carbon and free CaO content, all of which can be associated with the increased water demand [22,59]. This is also in agreement with [60], who investigated that the irregularly shaped particles form a very loose granular structure, resulting in a very high water demand.

Mixtures with binders based on WBA1, whose LOI content is higher, showed greater water demand compared to mixtures with WBA2 binders. The presence of organic matter (increased LOI values) favors a significant adsorption of water molecules, reducing the amount of free water required to achieve the desired workability [57,61].

Rapid loss of workability was observed in all mixtures but was less pronounced in the mixtures with higher CFA content. Bleeding was observed as early as 5 min after mixing and was particularly pronounced in mixtures containing WBA2. When mixed with water, WBA absorbs the water added to the mix, but when left to stand and mixed again, WBA releases water and gives a soft mortar [62]. Moderate bleeding is not a significant threat, as this property will increase the bond strength between the mortar and substrate [63]. Excess water will be absorbed by the masonry unit to improve the adhesion of the mortar.

The effect of WBA on setting time is shown in Table 6. The setting time increases with the increase in CFA content and can be explained by the slower reaction rate of CFA in the early stages [53,64]. The delayed setting time for blends with WBA1 can be directly correlated with the increased LOI content in these binders [65]. The deviation is observed only for the blend with 25% CFA and 75% WBA1. The setting could also be delayed by the higher content of alkalis (11.78%) in WBA1 binders compared to those with WBA2 (5.62%). The temperature of the paste could also contribute to the setting time. Free CaO is highly reactive [66] and leads to heat release [48], which could affect higher paste temperature. The temperature of the paste decreases with lower WBA content. According to [67], the low-calcium fly ash is often not self-cementing when mixed with water only, which could explain the prolonged setting time and lower temperature of the pastes with higher CFA content.

The volume stability was tested and it can be seen that the specimen with 75% content of WBA1 and the specimen with 100, 75, and 50% content of WBA2 exceed the criteria prescribed in EN 459-1:2015 [23], Table 6. Considering the whole series of specimens with WBA2, a magnified influence of the specific ash on the volume stability is also evident. These results are in agreement with the percentage of free CaO and free MgO determined for WBA2 as part of the research previously published by [19]. According to the research previously published by [19], it was found that the sample WBA1 had a higher percentage of free CaO and free MgO than the sample WBA2. Here, the storage time should also be considered, as WBA1 was collected 3 months before WBA2. This could lead to the stabilization of WBA1, so the mortar samples with WBA1 had better volume stability. The amount of expansive components such as free CaO and free MgO could be minimized due to various maturation processes (hydration, carbonation, oxidation), as well as insufficient fineness of the particles, which can be adjusted by grinding [32,68].

### 3.4. Influence of WBA and CFA Binder on Mortar Properties

The fresh state properties of the mortar with WBA and CFA binder are shown in Table 7. The amount of water selected was determined to ensure the required flow diameter (185 ± 3) mm according to EN 459-2 [30]. The presence of WBA caused an increased water requirement and reduced workability, as previously observed and explained for pastes. This is attributed to the morphological diversity of WBA and the high level of unburnt carbon (LOI) present in WBA. Thus, blends with WBA1 showed higher water requirements to obtain similar workability due to higher LOI values.

**Figure 7:**
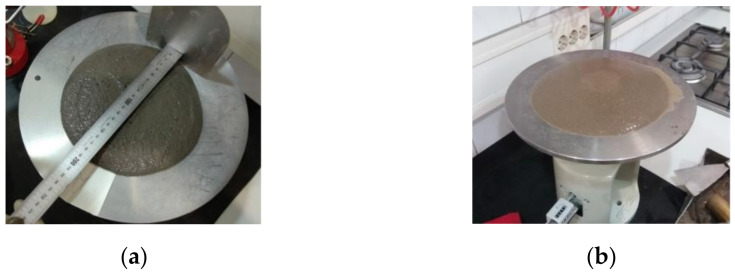
Flow table test: (**a**) WBA1, (**b**) WBA2.

The flow diameter test is shown in Figure 7. Here, bleeding was also observed in the mixtures with WBA2 which is probably caused by the lower content of fine particles in this WBA. Increased CFA content provided lower water requirement to achieve the desired workability grade.

Particle size distribution is the most important parameter affecting the bulk density of fresh mortar. Thus, the increase in finer particles of CFA causes an increase in mortar density proportional to the increase in their content. If the influence of WBA is examined, the filling effect of WBA1 is more pronounced due to its finer granulometry. This is consistent with previous investigations [69].

**Figure 8:**
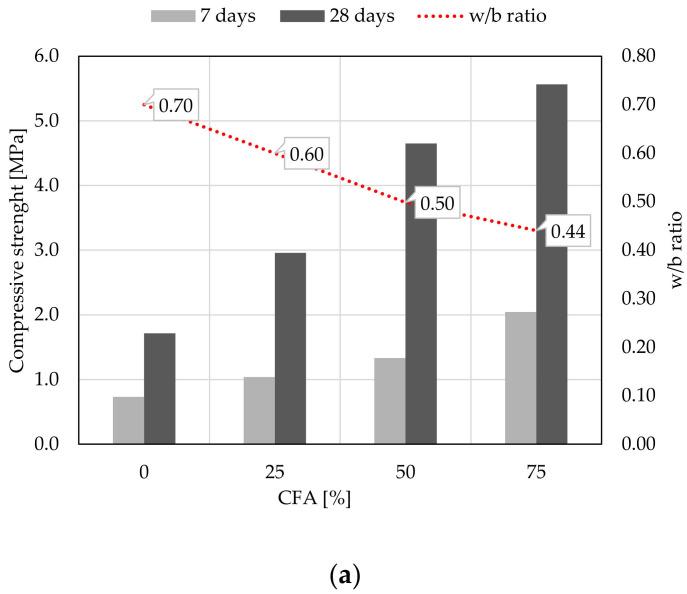

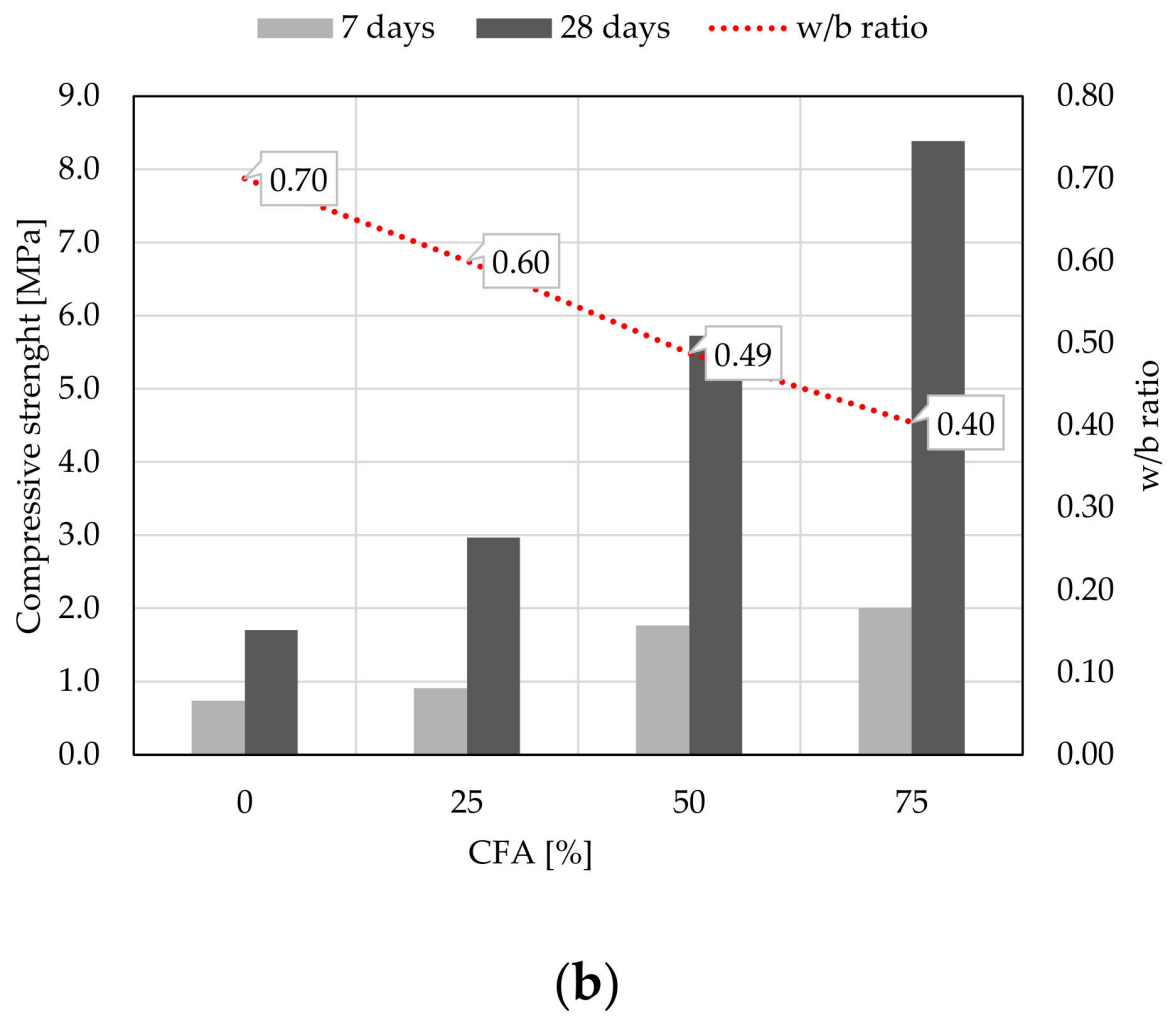
Compressive strength of mortars: (**a**) WBA1 and CFA binders, (**b**) WBA2 and CFA binders.

**Figure 9:**
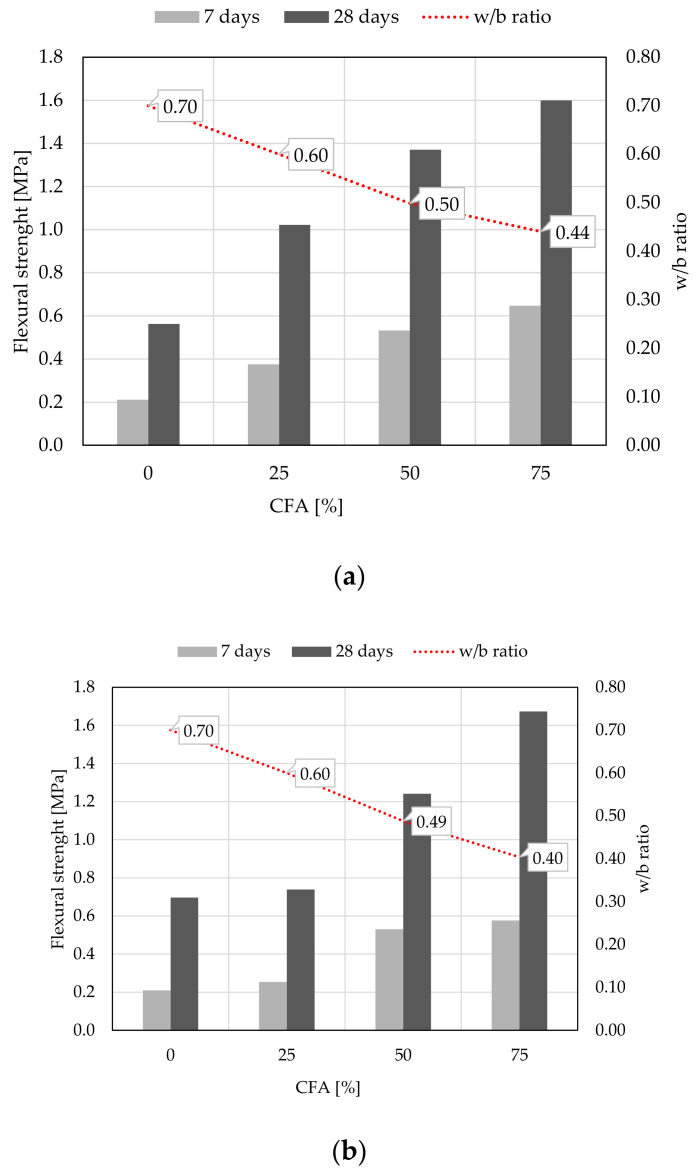
Flexural strength of mortars: (**a**) WBA1 and CFA binders, (**b**) WBA2 and CFA binders.

Compressive and flexural strength of mortar were determined at 7 and 28 days of age, Figure 8 and Figure 9. When analyzing the results of compressive strength for samples with 100% WBA content, the self-cementing property of this sample is noticeable, namely that WBA can set and harden when water is added [17,70,71,72,73]. After 7 days of specimen aging, the compressive strength is 0.74 MPa for WBA2 and 0.73 MPa for WBA1, or 1.71 MPa for both specimens after 28 days. Study [17] investigated the hydration of WBA and showed the development of 3CaO-Al_2_O_3_-Ca(OH)_2_xH_2_O (Afm), stratlingite (Ca_2_Al_2_ (SiO_2_)(OH)_10_ × 2.5(H_2_O)), CaCO_3_ and calcium silicate hydrates (C-S-H gel). The TGA results of the same study showed that a maximum portlandite content was formed after 3 days of hydration and decreased with further hydration, proving the pozzolanic activity of the ash [17]. Treatment of WBA with water showed that the main minerals during self-hydration are portlandite and calcite, with portlandite being converted to calcite with time of hydration [72]. During self-hardening, ettringite (Aft) and gypsum can be formed, and for ettringite formation, the aluminate content as Al_2_O_3_ of the ash should exceed the sulfur content and the pore solution (pH) should be above 11 [72], which is the case for the WBA2 sample. According to [67], the self-hardening strength is influenced by the chemical composition (especially the content of free CaO and SO_3_) and the fineness of the ash particles. These parameters are detected during the characterization of two different WBA samples. Although sample WBA2 has slightly higher portlandite content, lower SO_3_ content and coarser particles than WBA1, there is no difference in reactivity after 28 days between these two samples.

With further hydration, an increase in compressive strength results is seen for all specimens. The strength values for 28 days are 2.5 and 3 times higher than the results of compressive strength after 7 days, Figure 8. The difference in chemical composition and physical parameters of the used WBA is visible when testing mixtures with CFA; higher reactivity is observed for samples with 50 and 25% WBA2, and 50 and 25% CFA. A high LOI and alkali content [22,74,75,76,77] could lead to a reduction in compressive strength, as in the WBA1 samples. Despite the fact that commercial HL was not used in this study, the mechanical properties of the tested mortars with WBA gave excellent results after 28 days. The increase in both compressive and flexural strength is the result of the pozzolanic reaction of CFA and available Ca(OH)_2_, the reduced water to binder (w–b) ratio and the finer CFA granulometry, leading to an increase in mortar density, as previously shown by [69].

**Figure 10:**
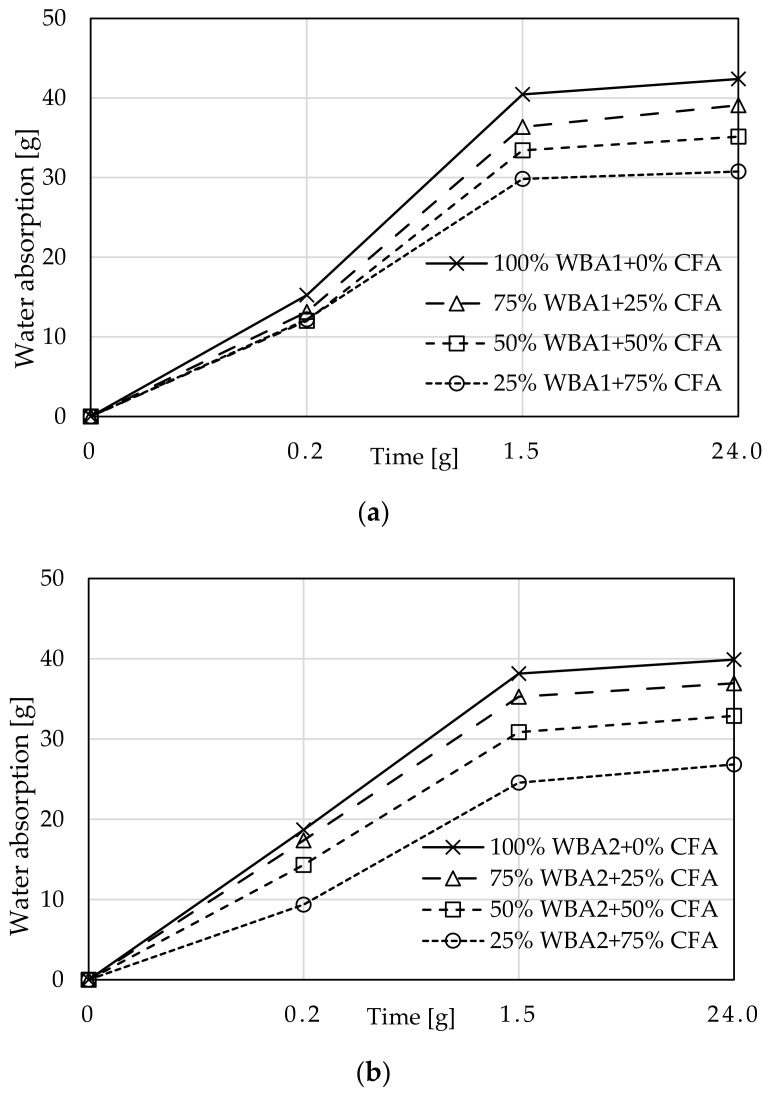
Capillary absorption of mortars: (**a**) WBA1 (**b**) WBA2.

**Figure 11:**
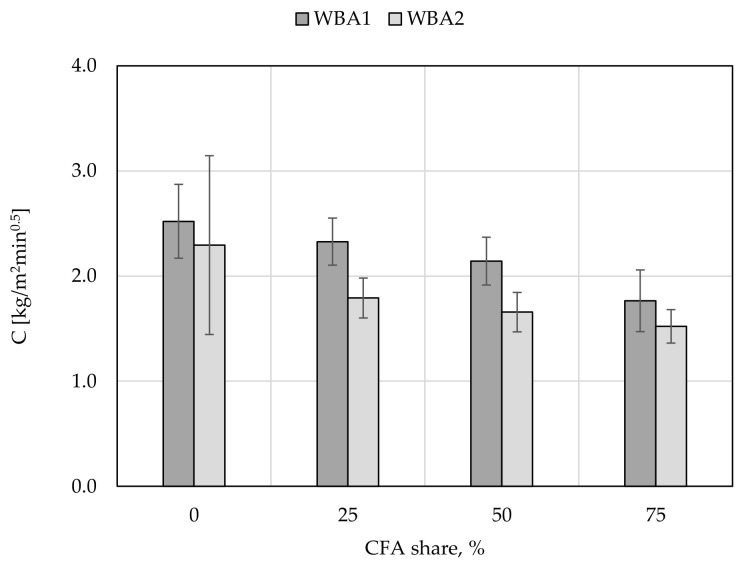
The coefficients of water absorption.

Capillary absorption was observed over time as a function of weight gain, Figure 10. All samples with WBA have a pronounced initial absorption over a period of 10 min, followed by a smaller weight gain until the next measurement after 90 min. After 90 min, water was visible on the top of the sample, indicating complete saturation. After 24 h, these samples were cut in half and water saturation was shown to be complete. With the increase in CFA content, decreased water absorption was demonstrated. These results are consistent with increased mortar density. Slightly better results exist for WBA2 mixes, which is also confirmed by the calculated water absorption coefficients for ordinary mortar (c_m_), Figure 11.

**Figure 12:**
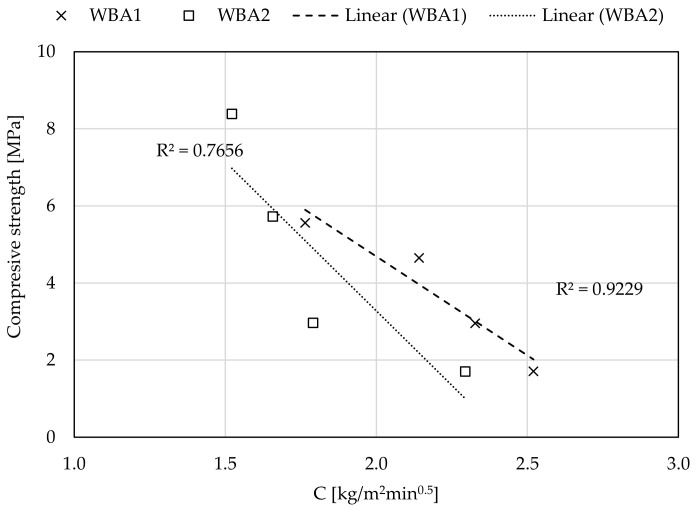
Correlation between the compressive strength and the capillary absorption coefficients.

The results for water absorption coefficients for ordinary mortars (c_m_) are 6–15% better for all WBA2 than for WBA1 samples, respectively. The study conducted by [38] on the cumulative pore volume and the average pore diameter showed that both slightly increased with the increasing amount of WBA in mortar, where most of the pores in lime-WBA systems were classified as capillary pores. In addition, according to [62], mortars with WBA have a higher volume of capillary pores, which are responsible for the higher water vapor permeability, open porosity, and highest total water absorption by capillarity. The obtained data for capillary absorption clearly confirm that mortars with higher percentage of WBA can affect the porosity of the microstructure, resulting in higher capillary absorption values. On the other hand, the addition of CFA improves the capillary absorption properties of mortars, i.e., reduces the value of water absorption coefficients. The plot of compressive strength and water absorption coefficients (Figure 12) shows a good correlation between these two properties: with higher CFA content in the WBA and CFA system, lower water absorption coefficients and higher compressive strengths can be expected. This results from a pozzolanic reaction between CFA and WBA, where pozzolanic oxides of CFA react with Ca(OH)_2_ formed by hydraulic reaction of free CaO of WBA and water. Therefore, an additional amount of C-S-H gel is formed, which thickens the mortar matrix, reduces the size of internal pores, and blocks capillary pores, resulting in a mortar with lower permeability [62].

#### Visual Appearance of the Specimens

**Figure 13:**
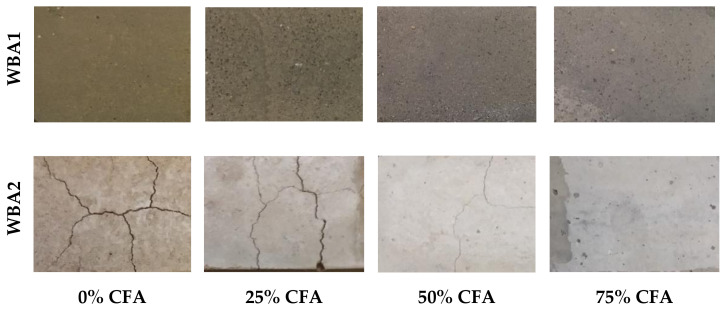
Surface appearance of samples with WBA1 and WBA2 after 28 days.

**Figure 14:**
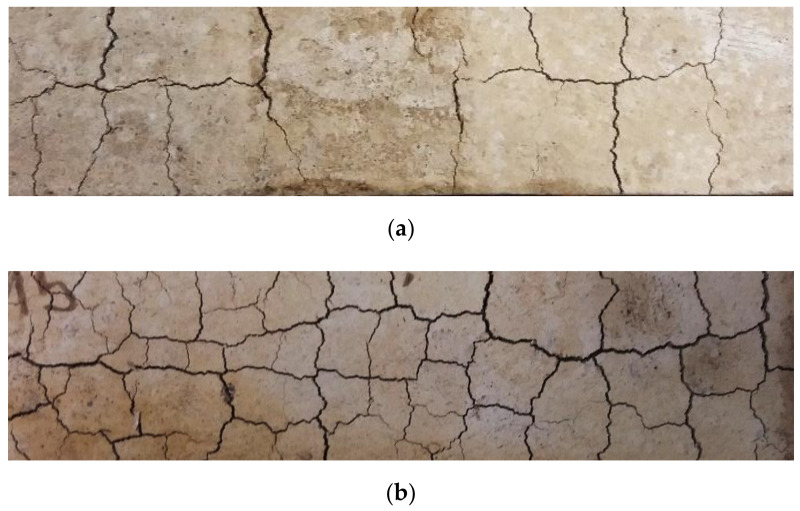
The surface appearance of samples cured in different conditions for WBA2: (**a**) 95% relative humidity (RH), (**b**) 65% relative humidity (RH).

The color of the specimens differs depending on the type and amount of WBA and CFA. Figure 13 shows the visual appearance of the WBA1 and WBA2 specimens. After the curing time, surface cracks appeared in the specimens with WBA2, while they were not observed in the WBA1 specimens. The number of cracks decreased with increasing CFA content, which is consistent with the results of the paste volume stability tests, which showed that WBA2 exhibits pronounced swelling. Specimens with 25% WBA2 did show visible cracks on the surface compared to the specimens with high WBA2 content. The occurrence of cracks was also influenced by the curing conditions. The reduced moisture content (60%) to which the specimens were exposed for the capillary absorption tests resulted in more cracks, Figure 14. 

This could be explained by the expansion of the Aft (ettringite) phase [67] that occurs during hydration [72]. Authors [78] reported that the extent of Aft-induced expansion depends on the curing conditions. In general, as water absorption from the environment increases, swelling is enhanced. However, expansion can also occur at a stage where the paste loses water through evaporation.

**Figure 15:**
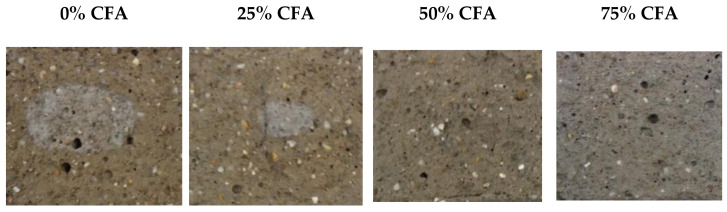
Internal appearance of samples with WBA1.

After the capillary absorption tests, the samples were split and patches of lighter color were noted in the center of the sample, being most pronounced in the samples without CFA and for WBA1, Figure 15. It should also be noted that an unpleasant odor was noted in the above samples after the capillary water absorption test was completed. The first assumption was that the color difference between the inner and outer part of the mortar was caused by the carbonation of the outer surface directly exposed to the atmosphere [79]. This could also explain the carbonation induced cracking [80] visible on the surface of the specimens, Figure 13 and Figure 14.

**Figure 16:**
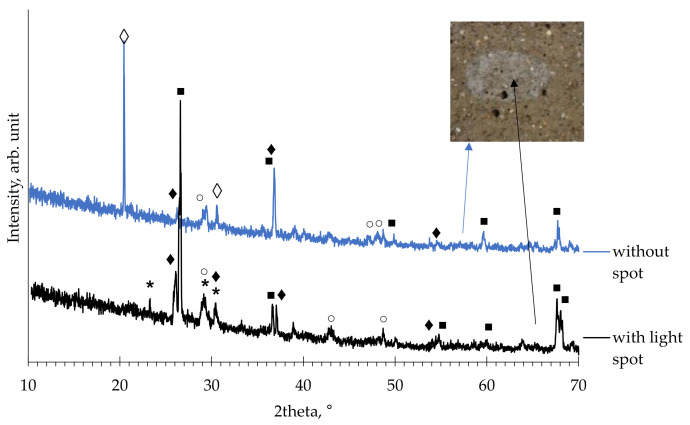
XRD pattern of split sample with 0% CFA (■—quartz; ○—calcite, *—CaAl_2_Si_3_O_10_×6H_2_O; ◊—Ca_2_Al_4_Si_8_O_24_∙12H_2_O; ♦—Ca_4_Fe_2_S_2_O_9_×12H_2_O).

In order to identified difference between phases of the inner (with light spot) and outer (without light spot) part, X-ray diffraction was used on slice of mortar. Diffractograms of the WB1 sample was obtained by X-ray diffraction (XRD) using a Bruker D8 Discovery diffractometer. Powdered samples were scanned between 10° and 70° with a step size of 0.02° using CuKα1,2 radiation. Immediately after cutting, a sample was put in isopropanol for 24 h to stop hydration (solvent exchange technique) and afterword XRD measurement was done. Results of hydrated sample of inner and outer part is given on Figure 16. The main phases are quartz, carbonate and calcium iron sulfate hydrate while difference between samples with and without light spot is connected to the formation of a different zeolitic phases: scolecite and laumontite as a hydrated calcium-aluminum silicate. No difference in the intensity of the carbonate phases was observed for inner and outer layer and therefor carbonation was not main cause of internal lighter color in WBA mortar sample. To fully understand WBA hydration and phases development, XRD analysis of mineral phases occurring at the different sample ages and different CFA content is proposed.

## 4. Conclusions

The presented research investigated the properties of binders, pastes and mortars with different proportions of WBA and CFA, aiming of classifying them as hydraulic lime according to HRN EN 459-1 and preparing an environmentally friendly mortar for the restoration of historic buildings. Based on the investigation performed, the following conclusions were drawn:

- After considering the chemical composition of WBA, the highest similarity with HL is found for WBA2 and then for WBA1, while WBA3 shows more similarity with coal fly ash. The particle size distribution shows a different trend starting with WBA1 as the finest to WBA3 as the coarsest material.
- A total of eight binders were prepared, four for each WBA (WBA1 and WBA2), as a mixture of WBA and CFA in the following ratios: 100:0, 75:25, 50:50, and 25:75. WBA3 was discarded due to its high content of silica and low content of available lime. The suitability of the tested binders was evaluated against the criteria prescribed in EN 459-1. All the binders tested met the specified criteria in terms of SO_3_ and available lime content. The particle size distribution criterion was met for all binders with WBA1, while for WBA2 only the binders with the lower content of WBA (WBA 50% + 50% CFA and WBA 25% + 75% CFA) met the criteria. The free water criterion was met for all binders tested.
- Pastes with produced binders were tested to check the conformity with the soundness and setting time criteria. The volume stability results indicate that the specimen with 75% content of WBA1 and the specimen with 100, 75, and 50% content of WBA2 exceed the criteria prescribed in EN 459-1:2015. Larger deformations were determined for the specimens with WBA2 what is in agreement with the percentage of free CaO and free MgO determined for WBA2. The setting time increased with the increase in CFA content and can be explained by the slower reaction rate of CFA and not self-cementing properties of the low-calcium fly ash when mixed with water only. The delayed setting time for blends with WBA1 can be directly correlated with the increased LOI and alkali content in these binders.
- The mortars with the prepared binders were tested for compliance with the air content and compressive strength criteria. All the tested mixes met the specified air content requirements, although slightly lower values, up to 6% on average, were obtained for the mixes with WBA1 due to the smaller particle size. The increase in both compressive and flexural strength of mortars with WBA and CFA binder compared to the HL after 28 days is the result of the pozzolanic reaction of CFA and available Ca(OH)_2_, the reduced w–b ratio and the finer CFA granulometry, leading to an increase in mortar density.

The comparison of the obtained results with the criteria of the HRN EN 459-1:2010 standard confirmed the possibility of classifying the binder with 25% content of WBA2 as HL5 and thus the possibility of using it as a renovation material. All other binders should be further optimized, paying special attention to the volume deformations that occur in pastes and mortars with higher contents of WBA2. The content of expansive components such as CaO, MgO, etc., could be canceled out by various maturation processes and insufficient fineness of the particles by grinding. WBA1 also shows potential for use in restoration mortars, as binders with 50% or less WBA1 meet all criteria except final setting time. This could be further improved by various chemical additives such as accelerators.

In conclusion, this new way of using WBA as a secondary raw material helps to make this waste, which is still landfilled, more environmentally friendly, resource efficient and recyclable. Reused together with CFA, which has a very similar fate, it provides a beneficial and improved source of revenue for biomass and power plants and a source of materials for the construction sector, as well as a means of reducing the carbon footprint of both.

## Figures and Tables

**Table 1:** Requirements for hydraulic lime (HL) according to standard EN 459-1 [23].

Properties			Requirements for HL		
			HL 2	HL 3.5	HL 5
Binder	SO_3_		≤3	≤3	≤3
	Available lime as Ca(OH)_2_		≥10	≥8	≥4
	Particle size (residue by mass (%))	0.09 mm	≤15	≤15	≤15
	Free water (%)		≤2	≤2	≤2
Pastes	Soundness	Alternative method (mm)	≤20	≤20	≤20
	Setting times [h]	Initial	>1	>1	>1
		Final	≤15	≤15	≤15
Mortars	Air content (%)		≤25	≤25	≤25
	Compressive strength [MPa]	7 days	-	-	≥2
		28 days	2 ≤ CS ≤ 7	3.5 ≤ CS ≤ 10	5 ≤ CS ≤ 15

**Table 2:** Characteristics of power plants where fly WBAs was collected.

		Combustion Properties		Biomass
Sample		Combustion type	Combustion temperature	Wood biomass type
WBA1	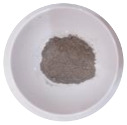	pulverized fuel combustion	700–750 °C	beech, oak, hornbeam and mixed wood
WBA2	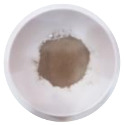	grate combustion	Up to 550 °C	beech, oak, abies and picea
WBA3	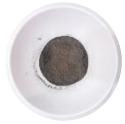	grate combustion	800 °C	beech, oak, hornbeam

**Table 3:** Test methods for characterization of wood biomass ash (WBA) and coal fly ash (CFA).

Property	Standard
Chemical composition	ISO/TS 16996:2015
pH value	EN 12176:2005
Available lime as Ca(OH)_2_	EN 459-2
Particle size (air jet sieving)	EN 459-2
Free water content	EN 459-2
Loss on ignition (LOI)	ASTM D 7348-13
TGA	-

**Table 4:** Test methods for characterization of paste and mortar properties.

Property	Standard	Number of Specimens (Per Mix)
Pastes		
Standard consistency	EN 196-3:2016	-
Setting time		-
Temperature		-
Soundness		3
Mortars		
Density	EN 1015-6:2000/A1:2008	-
Temperature	HRN U.M1.032:1981	-
Air content	EN 1015-7:2000	-
Consistence by flow table	EN 1015-3:2000/A1:2005/A2:2008	-
Compressive strength	EN 196-1:2016	3
Flexural strength	EN 196-1:2016	3
Capillary absorption	EN 1015-18:2002	3

**Table 5:** Chemical composition of WBAs and CFA used compared to HL [wt%].

	Non-Sieved WBA			Sieved WBA			CFA	Hydraulic Lime [23,40]
	WBA1	WBA2	WBA3	WBA1_s_	WBA2_s_	WBA3_s_		
pH value	13.5	13.15	12.87	13.48	13.18	12.99	12.50	-
P_2_O_5_	2.09	1.97	1.40	1.95	1.91	1.61	1.29	-
Na_2_O_eq_	15.3	5.9	5.59	11.78	5.62	5.72	3.7	-
CaO	43.68	57.93	18.58	49.33	56.53	23.80	6.67	58–60
MgO	3.92	6.17	3.68	3.7	6.74	4.27	2.26	-
Al_2_O_3_	2.53	3.14	12.42	2.86	3.43	10.62	29.00	2–7
TiO_2_	0.10	0.13	1.21	0.13	0.14	0.67	0.92	-
Fe_2_O_3_	1.71	2.10	4.79	2.01	2.26	4.32	7.09	1–3
SiO_2_	16.77	18.19	49.34	18.05	18.98	45.40	68.21	5–20
SO_3_	6.58	1.70	1.17	4.62	1.73	1.78	0.77	≤3
LOI	17.7	3.0	6.0	18.6	2.9	3.4	9.0	6–22
Ca(OH)_2_	n/a	n/a	n/a	19.90	22.85	7.03	0.80	≥10
								≥8
								≥4
From TG measurement								
Ca(OH)_2_	9.01	5.02	2.44	n/a	n/a	n/a	n/a	-
CaCO_3_	54.69	17.85	4.24	n/a	n/a	n/a	n/a	-

**Table 6:** Standard consistency, setting time and soundness of pastes.

	CFA Share (%)	Penetration (mm)	w/b Ratio	Temperature (°C)	Initial Setting Time (min)	Final Setting Time (min)	Soundness (mm)		
							A	B	(B-A)
WBA1	0	8	0.7	28.6	244	618	0.82	17.76	17
							3.26	20.07	17
							0	15.18	15
	25	8	0.53	24.9	33	94	0	20.76	21
							2.22	23.12	21
							1.1	20.19	19
	50	10	0.49	23.2	232	1375	2.54	5.58	3
							0	16.41	16
							0	13.24	13
	75	4	0.42	21.7	1401	1721	0	4.27	4
							1.68	14.32	13
							0.64	11.69	11
WBA2	0	6	0.60	29.7	97	142	0.8	27.84	27
							3.84	33.96	30
							1.87	33.15	31
	25	5	0.50	26.2	102	127	2.37	29.06	27
							3.14	29.23	26
							0	18.91	19
	50	4	0.45	-	332	402	0	12.13	12
							1.45	22.73	21
							0.77	19.96	19
	75	5	0.38	23.2	336	591	0.42	14.54	14
							2.77	12.3	10
							0	9.84	10

**Table 7:** Fresh state properties of mortars.

WBA Type	WBA Share (%)	CFA Share (%)	w/b Ratio	Temp. (°C)	Bulk Density (g/dm^3^)	Flow Diameter (mm)	Air Content (%)
WBA1	100	0	0.70	23.8	1898.1	181.5	4.8
	75	25	0.60	23.0	1941.6	185.5	4.6
	50	50	0.50	22.1	1962.3	185	4.6
	25	75	0.44	20.6	1992.7	182.5	4.8
WBA2	100	0	0.70	22.3	1887.2	184	5.0
	75	25	0.60	22.1	1893.4	193.5	5.4
	50	50	0.49	21.7	1940.4	185	5.2
	25	75	0.40	21.8	1982.9	195	4.4

## Data Availability

No new data were created or analyzed in this study. Data sharing is not applicable to this article.
